# Engineering of L-Plastin Peptide-Loaded Biodegradable Nanoparticles for Sustained Delivery and Suppression of Osteoclast Function In Vitro

**DOI:** 10.1155/2019/6943986

**Published:** 2019-05-05

**Authors:** Sunipa Majumdar, Aniket S. Wadajkar, Hanan Aljohani, Mark A. Reynolds, Anthony J. Kim, Meenakshi Chellaiah

**Affiliations:** ^1^Department of Oncology and Diagnostics, School of Dentistry, University of Maryland, Baltimore, USA; ^2^Departments of Neurosurgery and Pharmacology, School of Medicine, University of Maryland, Baltimore, USA

## Abstract

We have recently demonstrated that a small molecular weight amino-terminal peptide of L-plastin (10 amino acids; “MARGSVSDEE”) suppressed the phosphorylation of endogenous L-plastin. Therefore, the formation of nascent sealing zones (NSZs) and bone resorption are reduced. The aim of this study was to develop a biodegradable and biocompatible PLGA nanocarrier that could be loaded with the L-plastin peptide of interest and determine the efficacy* in vitro* in osteoclast cultures. L-plastin MARGSVSDEE (P1) and scrambled control (P3) peptide-loaded PLGA-PEG nanoparticles (NP1 and NP3, respectively) were synthesized by double emulsion technique. The biological effect of nanoparticles on osteoclasts was evaluated by immunoprecipitation, immunoblotting, rhodamine-phalloidin staining of actin filaments, and pit forming assays. Physical characterization of well-dispersed NP1 and NP3 demonstrated ~130-150 nm size, < 0.07 polydispersity index, ~-3 mV *ζ*-potential, and a sustained release of the peptide for three weeks. Biological characterization in osteoclast cultures demonstrated the following: NP1 significantly reduced (a) endogenous L-plastin phosphorylation; (b) formation of NSZs and sealing rings; (c) resorption. However, the assembly of podosomes which are critical for cell adhesion was not affected. L-plastin peptide-loaded PLGA-PEG nanocarriers have promising potential for the treatment of diseases associated with bone loss. Future studies will use this sustained release of peptide strategy to systematically suppress osteoclast bone resorption activity* in vivo* in mouse models demonstrating bone loss.

## 1. Introduction

During bone remodeling, mature bone tissue is removed by a process called bone resorption and new bone tissue is formed by a process called ossification or bone formation. Bone resorption and bone formation are mediated by osteoclasts and osteoblasts, respectively. Osteoclasts are multinucleated giant cells that resorb the inorganic and organic phases of bone (rev. in [1, 2]). During resorption, osteoclasts form a tight seal on the bone surface, onto which they secrete acid and proteases to facilitate the resorptive process [3–5]. This tight seal on the bone surface is associated with the formation of a ring of actin filaments known as sealing ring [6, 7].

Regulation of sealing ring formation in osteoclasts during bone resorption is a critical component in pathological bone loss. The regulation of sealing ring formation is dependent on the signaling mechanisms that regulate the interactions of actin-modulating protein(s) with actin filaments. Our recent studies have identified the assembly of a precursor zone (denoted as nascent sealing zones (NSZs)) at the early stage of sealing ring formation in active, bone-resorbing osteoclasts [6]. We demonstrated a novel mechanistic link between an actin-bundling protein L-plastin (LPL) and actin-binding protein cortactin in the formation of sealing rings [6]. LPL was shown to present in the podosomes of osteoclasts [8]. However, its function remains elusive in podosomes. Our previous and recent studies have shown that LPL has a regulatory role at the early phase of sealing ring formation in osteoclasts [6, 7, 9]

There are three isoforms of plastins (L-, T-, and I-plastin). Of the three, only L- and T-plastins regulate cytoskeletal reorganization via signal transduction pathways [10]. The L-plastin which was initially identified in leucocytes called “hematopoietic plastin isoform” (rev. in [11]) belongs to a large family of actin-crosslinking or bundling proteins, e.g., *α*-actinin and filamin [12]. Among the three plastins, only L-plastin has the capacity to bundle *β*-actin efficiently [13]. Plastin contains two phosphoserine residues (Ser-5 and Ser-7) and Ca2+binding sites flanked by EF-hand motifs at the amino terminal (NT) end. The C-terminal portion contains two tandem repeats of actin-binding domains, each of which consists of two sequential calponin-homology (CH) domains [14]. Of the three isoforms, only LPL has been shown to be phosphorylated in cells [10]. Phosphorylation of LPL on Ser-5 and Ser-7 amino acids (aa) was implicated in the cytoskeletal required for the processes of chemotaxis and cell adhesion [15–19].

We have previously shown that the phosphorylation of LPL on Ser-5 and Ser-7 aa by TNF-*α* signaling regulates the actin bundling process involved in the formation of NSZs in osteoclasts [6]. Subsequently, by transducing TAT-fused full-length LPL peptide (FL-LPL), we corroborated the role of LPL in the formation of NSZs. An increase in resorption in these osteoclasts corresponded well with an increase in the number of NSZs and sealing rings. Furthermore, the critical role of serine phosphorylation on the formation of NSZs and dentine resorption was demonstrated in osteoclasts transduced with a TAT-fused amino-terminal LPL (NT-LPL) peptides consisting of Ser-5 and Ser-7 aa. Transduction of NT-LPL peptide had the potential to reduce endogenous LPL phosphorylation which had an inhibitory effect on the formation of NSZs [7]. Furthermore, small molecular weight amino terminal LPL peptide (sNT-LPL; MARGSVSDEE; 10aa) containing Ser-5 and Ser-7 aa also demonstrated a similar significant inhibition of resorption by osteoclasts via attenuation of the formation of NSZs and sealing rings. But, bone formation by osteoblasts* in vitro* is unaffected by this sNT-LPL peptide. Substitution of the sNT-LPL peptide at Ser-5 and Ser-7 to Ala-5 and Ala-7 had no such inhibitory effects on osteoclast-mediated events [9]. Studies with TAT-fused sNT-LPL peptides suggest that LPL could be a novel target for treatment of bone loss without affecting the bone formation.

Biomaterials, especially polymeric nanoparticles, have been vastly investigated for a variety of applications including bone tissue engineering to enhance tissue regeneration and osseointegration of implants as well as to prevent infection [21–23]. Therefore, we explored the method of using polymeric nanoparticles to deliver and release sNT-LPL peptides of interest in a controlled and sustained fashion. Among different types of materials such as polymers, lipids, ceramics, and metals, polymers are of special interest for controlled and sustained drug delivery applications [24]. The most commonly used biocompatible and biodegradable polymers include poly (l-lactide) (PLA), polyglycolic acid (PGA), poly €-caprolactone (PCL), or the copolymer of PLA and PGA - poly (l-lactide-*co*-glycolic) (PLGA) [25, 26].

Bisphosphonates (BPs) are still one of the main therapeutic preferences for current treatment procedures of osteoporosis. Alendronate (aka Fosamax) is a bisphosphonate which is used in the treatment of bone loss in adults [27]. It was shown as a specific inhibitor of osteoclast sealing ring formation and activity in vitro [6, 28]. PLGA-alendronate (PLGA-ALE) nanoparticles were shown to disrupt sealing ring formation and induced apoptosis of osteoclasts. Therefore, inhibition of collagen degradation was observed. Overall, PLGA-ALE nanoparticles demonstrated antiosteoclastic activity [29–31]. However, it has been suggested that continued use of bisphosphonate therapy (e.g., alendronate) beyond the treatment period of 3 to 5 years may cause a reduction in osteoblast-mediated bone formation, resulting in atypical skeletal fractures in a patient who had used them [32–34]. Various risks associated with the use of BPs beside atypical fractures challenging for the development of novel antiresorptive drugs. Therefore, we believe that LPL is a novel therapeutic target and these preliminary observations with nanoparticles will provide justification for testing the nanoparticles in vivo in mice models.

Herein, the objective was to identify the application of sNT-LPL peptide-loaded PLGA nanoparticles on the actin remodeling processes involved in sealing ring formation and function in osteoclasts* in vitro*. Results from these studies suggest that nanoparticles formed from polymers may have appropriate targeting capability and deliver the peptides of interest in a sustained way in osteoclasts in vitro. Subsequently, this will enable the use of the PLGA nanoparticle preparation for the sustained systemic release of the peptide of interest over a period of days after administration into the mice, possibly circumventing everyday administration of the peptide by injection.

## 2. Materials and Methods

### 2.1. Materials

Methoxy terminated PLGA-PEG (10:5 kDa) was purchased from Polyscitech (West Lafayette, IN). Polyvinyl alcohol (PVA, 25 kDa) was purchased from Polysciences (Warrington, PA). Antibody to L-plastin (SC-16657) was purchased from Santa Cruz Biotechnology, Inc. (Santa Cruz, CA). Phosphoserine (125277) antibody was bought from Abcam (Cambridge, MA). Recombinant TNF-*α* (216-TA) and MCSF (416-ML) were purchased from R&D Systems (Minneapolis, MN). Protein estimation reagent, molecular weight standards for proteins, and PAGE reagents were bought from Bio-Rad. HRP-conjugated secondary antibodies for immunoblotting were obtained from GE Healthcare. PLGA (7-17 kDa, 50:50), dichloromethane (DCM), anti-rabbit antibodies to GAPDH, Rhodamine-phalloidin, and other chemicals were purchased from Sigma-Aldrich (St. Louis, MO). sNT-LPL peptides (fluorescence-conjugated and unconjugated) are made from Genscript Co. (Piscataway (NJ)).

### 2.2. Synthesis of Nanoparticles

Peptide-loaded nanoparticles were synthesized by double emulsion solvent evaporation technique. Briefly, the organic phase was prepared by dissolving the polymers (14 mg PLGA and 14 mg PLGA-PEG) in 2 ml DCM. The primary water-in-oil emulsion was formed as follows: under vigorous stirring, aqueous peptide (P1 or P3) solution (50 *μ*l, 10 mM) was added dropwise to the polymer solution. PVA (5% w/v) was dissolved in water and passed through a 0.2 *μ*m filter to form the secondary water phase. After 30 min stirring, the primary emulsion was added to the aqueous PVA solution (12 ml) to form the water-in-oil-in-water emulsion. Sonication was done immediately in an ice bath using ultrasonication probe (Sonics Vibra-Cell, Newton, CT) at 30% amplitude for 2 min with 20-sec on-off pulses. After the sonication, the emulsions were transferred to magnetic stirring for 4 h at room temperature to allow DCM to evaporate. Nanoparticles (NPs) formed (PLGA-PEG_P1 and PLGA-PEG_P3 peptides; henceforth denoted as NP1 and NP3) were subjected to three washes by microcentrifugation at 21,100 ×* g* for 10 min. Subsequently, NPs were resuspended in ultrapure water and used fresh for experiments [20].

### 2.3. Characterization of Nanoparticles

NPs were diluted 15X with PBS (~10 mM NaCl, pH 7.4) prior to the physicochemical characterization. Hydrodynamic diameter, polydispersity index (PDI), and *ζ*-potential (surface charge) were determined by dynamic light scattering and laser Doppler anemometry using Zetasizer NanoZS (Malvern Instruments, South Borough, MA). Particle size measurements were performed at 25°C at a scattering angle of 173° and are reported as the number-average mean. The surface charge on the particles was reported as the mean *ζ*-potential. The structure and morphology of NP were observed using a FEI Tecnai T12 transmission electron microscope (TEM, FEI, Hillsboro, OR) operated at 80 kV [20].

### 2.4. Peptide Loading and Release

For peptide loading measurements, FITC-conjugated sNT-LPL peptides of interest were loaded in the nanoparticles following the same double emulsion method mentioned above. After nanoparticle preparation, 1 mg lyophilized nanoparticles were dissolved in 0.2 ml DCM, to which 0.5 ml water was added, vortexed, and set aside for phase separation. The water phase containing peptides was carefully collected from the top without disturbing the bottom phase. The fluorescence of the collected peptide solutions was analyzed by the spectrometer at 495 nm excitation and 520 nm emission wavelength. For peptide release study, known amounts of lyophilized nanoparticles were suspended in PBS and placed in Float-A-Lyzer dialysis tubes (3.5–5 kDa MW cut-off, Spectrum Labs, Rancho Dominguez, CA). The samples were dialyzed against PBS on an orbital shaker at 37°C. At predetermined time intervals, 1 ml dialysate was collected, and the same volume was replenished with fresh PBS incubated at 37°C. All the collected peptide release samples were analyzed on a spectrometer with the wavelength settings mentioned above. The peptide concentrations were quantified from a standard curve created by reading fluorescence intensities of serially diluted known peptide concentrations. The peptide release % was calculated from the following equation:

Peptide release (%) = (amount of peptide in dialysate × 100) / (amount of peptide in nanoparticles inside dialysis tube) [20].

### 2.5. Preparation of Osteoclast Precursors from RAW264.7 Macrophage-Like Cell Line

Murine osteoclasts were generated from RAW264.7 cells as described [35]. Briefly, RAW264.7 cells were plated at a low density in the presence of Dulbecco's modified Eagle's medium (DMEM) with 10% fetal bovine serum. After 24 h, cells were treated with macrophage colony-stimulating factor (M-CSF; 10 ng/ml) and GST-RANKL (60 ng/ml). After two days, the medium was replaced with fresh M-CSF and RANKL at indicated concentrations above. Recombinant GST-RANKL was purified as described previously [6]. Mature multinucleated osteoclasts were seen from day three onwards.

### 2.6. Lysate Preparation after the Addition of Osteoclasts with Peptides and Bone Particles

Preparation of mouse bone particles and the addition of bone particles to mature osteoclasts were done as described previously [6]. Three to six wells were used for treatment with nanoparticles loaded with peptides or transduction with TAT-fused sNT-LPL peptides and lysate preparation. For transduction with TAT-fused peptides or treatment with nanoparticles, osteoclasts were first kept in serum-free DMEM for two 2 h. TAT proteins (100-150 nM) or nanoparticles (150 nM) of interest were added to cultures for 2 h in serum-free DMEM. Subsequently, bone particles (60-80 *μ*m size; 100 *μ*g/well) and TNF-*α* (20 ng/ml) were added to cells and incubation was continued for 4 h or 6 h depending on the experimental conditions.

Following various treatments, after three washes in ice-cold PBS, osteoclasts were lysed in RIPA buffer (10 mM Tris- HCl, pH 7.2, 150 mM NaCl, 1% deoxycholate, 1% Triton X-100, 0.1% SDS, 1% aprotinin, 2mM PMSF, 100 M Na_3_VO_4_, and 1% aprotinin) and centrifuged at 15,000 rpm for 5 min at 4°C. The supernatant was used for protein estimation using Bio-Rad protein assay reagent.

### 2.7. Preparation Immunoprecipitation and Immunoblotting Analysis

Equal amounts of lysate proteins (100-150 *μ*g) were used for immunoprecipitations. Immunoprecipitations and Western blotting were performed as described previously [36, 37].

### 2.8. Rhodamine-Phalloidin Staining of Actin Filaments in Osteoclasts

Osteoclasts were generated from RAW 264.7 macrophage-like cell line as described above. For staining of podosomes, RAW cells were plated on glass coverslips and differentiation was done as described above with M-CSF and RANKL. Mature multinucleated osteoclasts were seen from day three onwards. Undifferentiated RAW cells were gently removed with cell stripper solution (Sigma), and osteoclasts were either transduced with TAT-fused peptides or treated with NPs in the presence of TNF-*α* and bone particles. To detect NSZs and sealing rings, mature osteoclasts were removed with cell stripper solution and replated on dentine slices. Cells were allowed to attach to a mineralized matrix for four to five hours; then cells were either transduced with TAT-fused peptides or treated with NPs in the presence of TNF-*α*. Incubation was continued for 3-4 h to detect NSZs or 12-14 h to detect sealing rings [38]. Actin staining was done in these osteoclasts with rhodamine-phalloidin as described [38]. Stained osteoclasts were photographed with a Bio-Rad confocal laser-scanning microscope. Images were stored in TIF image format and processed by Adobe Photoshop (Adobe Systems Inc., Mountain View, CA).

### 2.9. Resorption Pit Formation Assay* In Vitro* Using Dentine Slices

Resorption assay was performed as previously described [39]. To determine resorption efficiency, osteoclasts replated on dentine slices were allowed to attach to a mineralized matrix for four to five hours. Subsequently, osteoclasts were either transduced with TAT-fused peptides or treated with NPs in the presence of TNF-*α*. Incubation was continued for 12-14h to detect resorption pits [38]. Resorbed areas were scanned in Bio-Rad confocal microscopy. Images were stored in TIF format and processed by Adobe Photoshop (Adobe Systems Inc.).

### 2.10. MTT Assay for Viability Measurements

Cell viability was assayed by measuring blue formazan formed from the cleavage of 3-(4-5-dimethlthiazol-2-yl) 2-5-diphenyl tetrazolium bromide (MTT)) salt by the mitochondrial dehydrogenase enzyme (Sigma). Differentiated osteoclasts treated with nanoparticles (NP1 and NP3; 150nm) or transduced with TAT-fused peptides (P1 and P3; 150nm)) for 4h were subjected to viability assay. MTT was added to each well and incubated for 2h at 37°C. To stop the chemical reaction, an MTT solubilization solution provided by the manufacturer was added to the wells. Subsequently, the plate was read at 570 nm as per instructions provided in the protocol by the manufacturer (Sigma). Background values from empty wells were subtracted, and data normalized to osteoclasts untreated with any peptide to peptide treated osteoclasts. Three to four wells were used for each treatment. Statistical significance was measured as described below.

### 2.11. Tartrate-Resistant Acid Phosphatase (TRAP)-Staining

Briefly, cells were untreated or treated with peptides of interest for 4h, subsequently fixed with 4% paraformaldehyde, and then washed three times with PBS. TRAP staining was done with Leukocyte Acid Phosphatase Kit (Sigma; 387-A) according to the protocol provided by the manufacturer. Stained cells were photographed with phase contrast microscopy, and images were processed in Adobe Photoshop (Adobe Systems Inc.)

### 2.12. Statistical Analysis

We used either one-way or two-way ANOVA followed by multiple comparisons tests to determine significance (Graph Pad Software, Inc., La Jolla, CA). All values presented as mean ± SEM. A value of p < 0.05 was considered significant.

## 3. Results

We have recently shown that transduction of TAT-fused small molecular weight amino-terminal L-plastin peptides (10 amino acids; “1MARGSVSDEE10” ) containing phospho- Ser-5 and Ser-7 has the potential to suppress the phosphorylation of endogenous LPL competitively and hence NSZs formation and resorption by osteoclasts [9]. Next, to determine an efficient uptake and release of the peptide of interest, we utilized nanoparticles loaded with sNT-LPL peptides (NP1 and NP3) of interest in osteoclast derived from RAW 264.7 murine cell line. RAW cell line derived osteoclasts have been widely used for several in vitro studies due to their availability and simple culture system [35, 40–44].

### 3.1. Analyses in RAW 264.7 Murine Cell Line Derived Osteoclasts with sNT-LPL Peptides

#### 3.1.1. Time-Dependent Uptake of sNT-LPL Peptides

**Figure 1:**
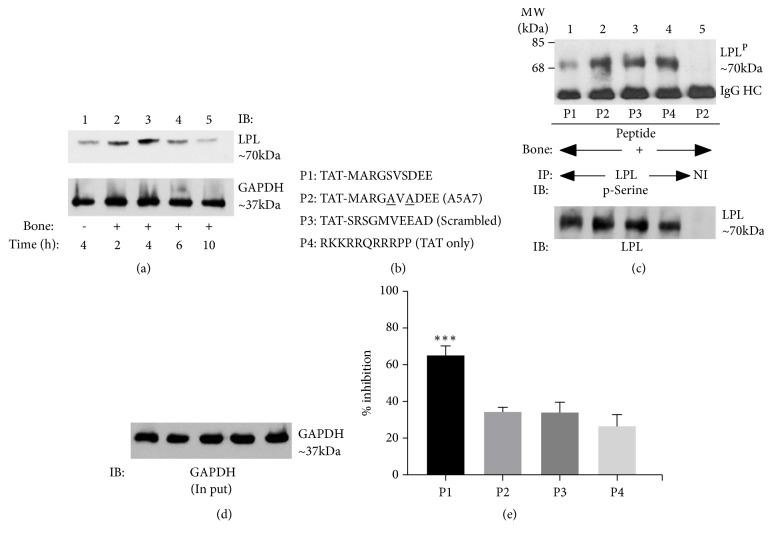
Immunoblotting analyses with an L-plastin (LPL) and a p-Serine antibody. (a) Analysis of time-dependent expression of LPL in osteoclasts treated with (+; lanes 2-5) and without (-; lane 1) bone particles in the presence of TNF-*α*. (a) Immunoblotting analysis with an antibody to LPL (top panel) and GAPDH (bottom panel). (b) Amino acid sequences of TAT (11aa) -fused sNT-LPL (10aa) and control TAT alone (11aa) peptides are shown: P1) unsubstituted (S5S7); P2) double substituted (S5S7 to A5A7); P3) scrambled; P4) control TAT sequences only. (c). Immunoprecipitation and immunoblotting analyses. The effect of indicated sNT-LPL peptides (P1 to P4) on the phosphorylation of endogenous LPL is shown. An equal amount of osteoclast lysates was immunoprecipitated with an antibody to LPL and subjected to immunoblotting (IB) with a p-Serine antibody (top). This blot was stripped and blotted with an LPL antibody (panel (c); middle). Immunoprecipitation of lysates made from P2-transduced cells with a species-specific nonimmune serum was used as a control for immunoprecipitation (lane 5). An equal amount of total protein (Input) used for immunoprecipitation was assessed by direct immunoblotting of lysates with a GAPDH antibody. The experiment was repeated thrice and obtained comparable inhibitory effect with P1.

We first characterized whether RAW cell-derived osteoclasts exhibit similar characteristics as osteoclasts derived from mouse bone marrow in response to bone particles and TNF-*α* [6, 7]. RAW cell-derived osteoclasts also displayed a time-dependent increase in LPL levels at 2-4 h (Figure 1(a), Lanes 2 and 3) and decreased thereafter (Lanes 4 and 5).

#### 3.1.2. Amino Acid Sequences of TAT-Fused sNT-LPL Peptides

The amino acid (aa) sequences of following TAT-fused small molecular weight amino-terminal -LPL peptides (sNT-LPL; 10aa) are shown in Figure 1(b): P1: peptide containing phospho- Ser-5 and Ser-7; P2: Ser-5 and Ser-7 are substituted with Ala-5 and Ala-7; P3: scrambled peptide; P4: peptide containing TAT sequences only (11 aa).

#### 3.1.3. sNT-LPL Peptide P1 Reduces the Phosphorylation of Endogenous LPL

We then confirmed whether the sNT-LPL-P1 peptide has the potential to inhibit endogenous LPL phosphorylation in RAW cell-derived osteoclasts (Figure 1(c)). Osteoclasts were transduced with indicated TAT-fused peptides for 4 h and lysates were immunoprecipitated with an antibody to LPL (Figure 1(c), Lanes 1-4). Lysate made from osteoclasts transduced with P2 peptide was immunoprecipitated with a species-specific nonimmune serum (NI, Lane 5). Immunoprecipitates were immunoblotted with a p-serine antibody (Figure 1(c), Top). A significant inhibitory effect on the phosphorylation of endogenous LPL was observed in osteoclasts transduced with P1 peptide (Figure 1(c); Lane 1) as compared with control peptides P2-P4 (Figure 1(c); Lanes 2-4). The inhibition with P1 peptide was found to be greater than 60% as compared with controls (P2-P4) (Figure 1(e)). The levels of LPL protein in each immunoprecipitate are shown after stripping and reblotting with an antibody to LPL (bottom panel of Figure 1(c)). Immunoblotting of total lysates with an antibody to GAPDH validates that an equal amount of protein was used for immunoprecipitation (Figure 1(d)).

These observations corroborate our previous studies in mouse osteoclasts [7, 9]. Therefore, we used RAW cell-derived osteoclast system to further confirm the effects of LPL peptide-loaded PLGA-PEG nanoparticles (NP1 and NP3) on endogenous LPL phosphorylation, osteoclast actin remodeling processes involved in the formation of NSZs, sealing rings, and resorption pits. We used scrambled peptide-loaded nanoparticle (NP3) as a control.

### 3.2. Physicochemical Characterization of LPL Peptide-Loaded PLGA-PEG Nanoparticles

**Figure 2:**
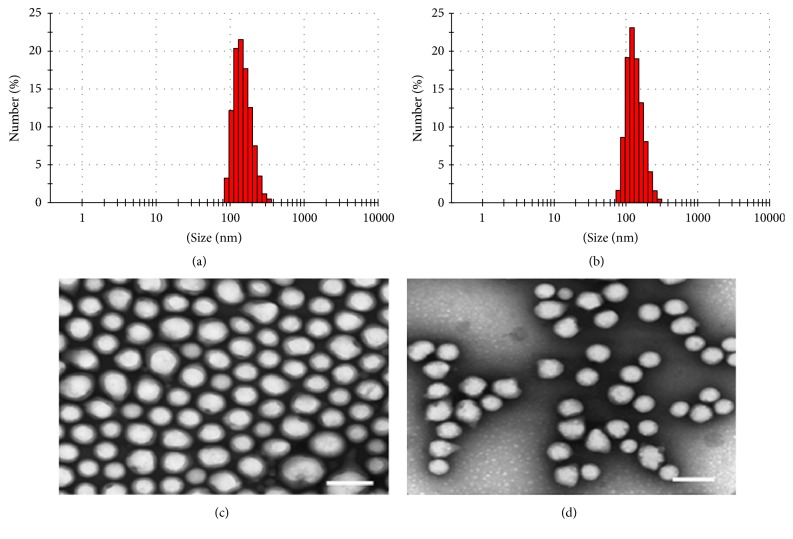
*Nanoparticle size distribution and morphology*. A narrow size distribution of PLGA-PEG_P1 (a) and PLGA-PEG_P3 (b) nanoparticles measured by dynamic light scattering is shown. (c) and (d) Transmission electron microscopy (TEM) images show well-dispersed round shaped PLGA-PEG_P1 (c) and PLGA-PEG_P3 nanoparticles (d). Scale bars = 200 nm.

L-plastin peptide-loaded PLGA-PEG nanoparticles (NP1 and NP3) were synthesized by double emulsion technique as described previously [20]. The hydrodynamic diameter of the nanoparticles measured by dynamic light scattering was 157 ± 4 nm and 136 ± 5 nm for NP1 and NP3, respectively (Table 1). PDI of 0.058 and 0.062 (Table 1), as well as a narrow size distribution (Figures 2(a) and 2(b)) in dynamic light scattering analyses, indicate that the nanoparticles were well dispersed and stable in the solvent. In addition, imaging of nanoparticles by TEM confirmed the size of the nanoparticles as well as round particle morphology (Figures 2(c) and 2(d)). The difference between the two TEM images is due to different concentrations of the nanoparticles in the samples. Moreover, the zeta potential of the nanoparticles was measured in terms of surface charge. Both the nanoparticle types had nearly neutral charge of -3.6 mV and -3.1 mV, respectively (Table 1).

### 3.3. Peptide Loading and Release from Nanoparticles

**Figure 3:**
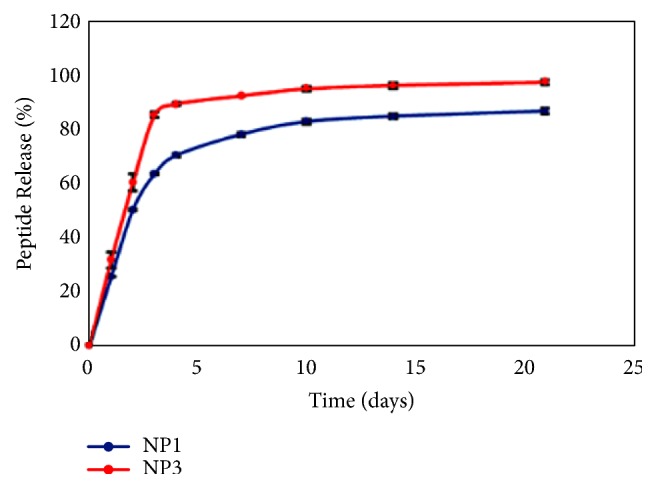
*In vitro release profile of peptide from PLGA-PEG nanoparticles*. Graphical representation of in vitro release of PLGA-PEG_P1 and PLGA-PEG_P3 plotted as a function of percent peptide release versus days from PLGA-PEG nanoparticles. The graph represents one of the two experiments performed.

Peptide loading efficiency was evaluated by measuring fluorescence from the FITC-conjugated peptides. We estimated ~5% w/w loading of peptides in the nanoparticles (Table 1). Peptide release study was then performed to quantify the release profiles of peptides from the nanoparticles. We observed biphasic release profiles of both the peptides with a burst release during the initial three days followed by a slow and sustained release over a period of three weeks (Figure 3). We also observed a higher and complete (100%) release of P3 peptide compared to that of P1.

### 3.4. Analysis of the Effect of NP1 and NP3 on Endogenous L-Plastin Phosphorylation

**Figure 4:**
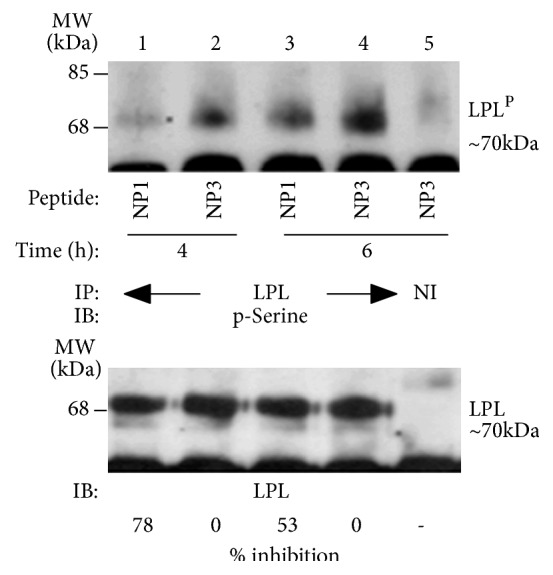
*Immunoblotting (IB) analysis of the time-dependent effect of NP1 and NP3 on the phosphorylation of endogenous LPL*. The equal amount of lysates made from osteoclasts treated with NP1 (lanes 1 and 3) and NP3 (lanes 3 and 4) for 4h (lanes 1 and 2) and 6h (lanes 3 and 4) was used for immunoprecipitation (IP) with an LPL antibody. IP with a nonimmune serum is shown in lane 5. Immunoprecipitates were subjected to IB with an antibody to p-Serine (top panel). Blot was stripped and reprobed with an antibody to LPL (bottom panel). Percent inhibition of phosphorylation of the representative experiment with NP1 is 78% at 4h and 53% at 6h as compared with corresponding NP3 control in lanes 2 and 4. These results represent one of the two experiments performed with similar results.

We then determined the effects of NP1 and NP3 on the phosphorylation of endogenous LPL in the presence of bone particles and TNF-*α* for 4 h and 6 h (Figure 4). Lysates made from these osteoclasts were immunoprecipitated with an LPL antibody (Figure 4, Lanes 1-4) or nonimmune serum (NI, Lane 5). Immunoblotting was done with a p-serine antibody. As shown in Figure 1(c) with P1 peptides, NP1 demonstrated a significant inhibitory effect on the phosphorylation of endogenous LPL at 4 h and 6 h (Figure 4, lanes 1 and 3) as compared with NP3 (Lanes 2 and 4). The inhibition was more at 4h (78%) than 6h (53%) as compared with respective NP3 controls in lanes 2 and 4 (Figure 4; top panel). The levels of LPL protein in each immunoprecipitate are shown after stripping and reblotting with an antibody to LPL (Figure 4; bottom panel). The experiment was repeated twice and obtained a comparable inhibitory effect with NP1 at 4 h and 6 h. These studies evaluated the efficiency of PLGA-PEG nanoparticles (NP1) in the inhibition of phosphorylation of endogenous LPL protein. These studies suggest that PLGA-PEG nanoparticles could be used as a possible peptide delivery carrier for the inhibition of osteoclast function.

### 3.5. Analysis of Cell Viability by MTT Assay and TRAP-Staining

**Figure 5:**
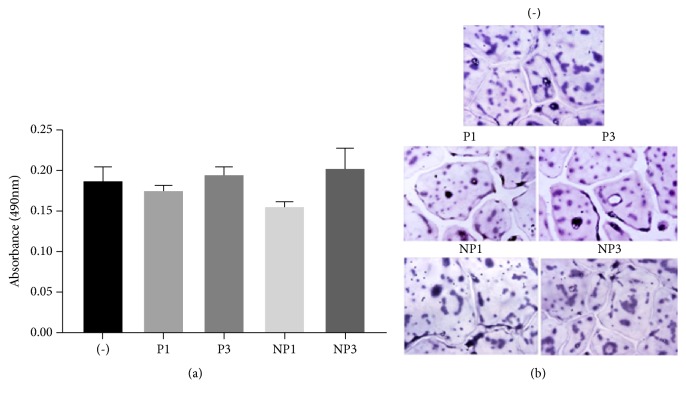
*MTT assay and TRAP-staining of osteoclasts after treatment with TAT-fused sNT-LPL peptides (P1 and P3) and nanoparticles (NP1 and NP3).* Osteoclasts were treated with indicated peptides or nanoparticles for 4h in the presence of TNF-*α* and bone particles. Next, cells were subjected to the calorimetric MTT assay as described in the Methods to determine the proliferation effects (a). TRAP staining was performed to determine the cell morphology or phenotype of osteoclasts (b). Osteoclasts untreated with peptides but treated with TNF-*α* and bone particles were used as controls (-). MTT assay confirmed that proliferation activity is not affected. Morphological characterization by TRAP-staining and attachment of osteoclasts to culture dishes suggest that cells are active and viable. Magnification-40X. These results represent one of the two experiments performed with similar results.

We also determine the viability of cells by MTT assay and TRAP-staining at 4h after treatment with and without peptides. After differentiation of RAW 264.7 cells into multinucleated bone-resorbing osteoclasts, the excess undifferentiated RAW cells were gently removed using cell stripper solution (Sigma). Subsequently, multinucleated osteoclasts with little or no RAW cells were treated as indicated in Figure 5 in the presence of bone particles and TNF-*α* for 4h. After treatment for 4h, cells were either assayed for cell viability by MTT assay (Figure 5(a)) or subjected to staining for TRAP enzyme which is a marker for osteoclasts (Figure 5(b)). MTT assay and TRAP staining demonstrated that viability or morphology was not affected by any of the peptide treatment (Figure 5(a)). Well-spread multinucleated mature osteoclasts positive for TRAP enzyme are shown (Figure 5(b)). Untreated cells (-) demonstrated similar characteristics on viability and morphology to the cells treated with indicated peptides or nanoparticles.

### 3.6. Analysis of the Effects of Peptide (P1 and P3) and Nanoparticles (NP1 and NP3) on Actin Modulation In Vitro

**Figure 6:**
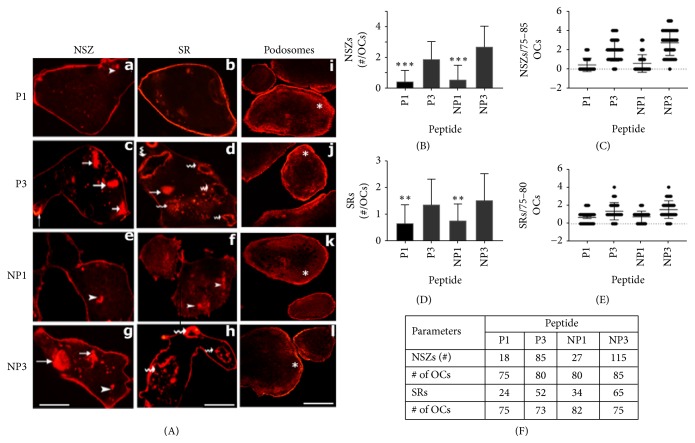
*Confocal microscopy analyses of osteoclasts stained for filamentous (F-) actin with phalloidin*. (A) Actin staining of osteoclasts with rhodamine phalloidin: osteoclasts were transduced with TAT-fused sNT-LPL peptides (P1—panels a, b, & I; P3—panels c, d, & j) or treated with nanoparticles loaded with peptides (NP1—panels e, f, & k; NP3—panels g, h, & l). After treatment cells were stained for actin (red) to determine the formation of nascent sealing zones (NSZs) and sealing rings (SR). Arrowheads point to actin punctate stainings; arrows point to NSZs; wavy arrows point to mature sealing rings. Osteoclasts plated on coverslips and treated as indicated above with peptides, and bone particles were stained with rhodamine phalloidin. An asterisk indicates podosome localization at the cell periphery. Scale bar 150*μ*m. These results represent one of the three experiments performed with similar results. (B)-(F) Statistical analyses of the number of NSZs and sealing rings (SRs). The number of NSZs and SRs was counted in 75-80 osteoclasts from three experiments and presented as graphs (B=E) and table  (F). The treatments are shown below each graph. The number of NSZs and SRs is presented per osteoclasts in graphs shown in (B) and (D). Data are also given as scatterplots for the indicated number of osteoclasts ((C) and (E)). The total number of NSZs and SRs is also provided for the indicated peptides and the number of osteoclasts in Table  (F). The effect of P1 or NP1 is significant in the inhibition of NSZs and SRs as compared with respective control groups (P3 or NP3). *∗∗*p<0.01; *∗∗∗*p<0.001 versus respective control groups (P3 or NP3).

To further define and highlight the impact of serine phosphorylation on actin bundling process, osteoclasts plated on the mineralized matrix (osteologic discs) and treated with NP1 and NP3 for 4 h and 12 h were stained for actin using rhodamine phalloidin. Four and 12h treatments were used to determine the inhibitory effect of NP1 on the formation of NSZs and sealing rings, respectively (Figure 6). We used Tat-fused peptides (P1 and P3) as controls for NP1 and NP3 (Figure 6(A)). Osteoclasts transduced with peptide P1 demonstrated a significant decrease in the number of NSZs and sealing rings (Figure 6(A); panels a and b) as compared with osteoclasts transduced with peptide P3 (panels c and d). NP1- and NP3-treated osteoclasts (Figure 6(A); panels e-h) corroborated the results observed with P1 and P3 (Figure 6(A); panels a-d), respectively. The number of NSZs and sealing rings was counted in about 75-85 osteoclasts and are provided as graphs and table in Figure 6 (panels (B)-(F); n=3). The number of NSZs and sealing rings is considerably decreased in osteoclasts transduced with P1 or treated with NP1. This inhibition corresponds with a significant inhibitory effect on the phosphorylation of endogenous LPL (Figure 4, top panel, lane 1). As shown previously [9], these peptides did not affect the organization of podosomes in osteoclasts plated on glass coverslips and treated with (P1 and P3) or nanoparticles (NP1 and NP3) (Figure 6(A), panels i-l).

### 3.7. Analysis of Resorption Activity Using Dentine and Resorption Markers by Immunoblotting

**Figure 7:**
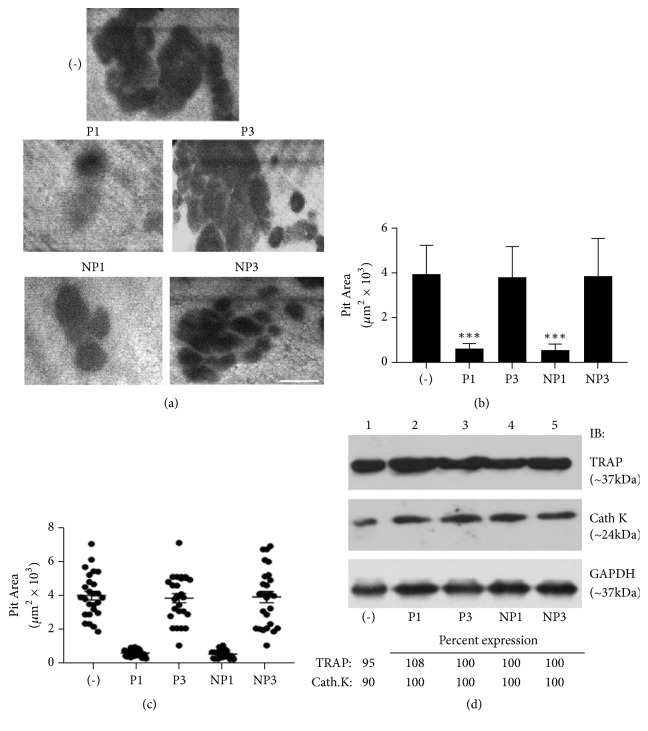
*Resorption assay using dentine matrix and immunoblotting analyses for resorption markers*. Analysis of the effects of transduced TAT-LPL peptides (P1 and P3) and nanoparticles (NP1 and NP3) on resorption by osteoclasts using dentine slices. (a) Osteoclasts were cultured on dentine slices for 10–12h in the presence of TNF-*α* and indicated peptides. Osteoclasts untreated with any peptide but treated with TNF-*α* were used as controls (-). Pits were scanned in a Bio-Rad confocal microscopy. Scale bar- 25*μ*m. Resorbed areas are seen as dark areas. (b) and (c) Statistic measurements of the pit area are provided as a graph: *∗∗∗*p < 0.001 versus respective controls (P3 or NP3). The resorbed pit areas (8-10 pits/slice) were quantified, and data were compiled from three dentine slices per treatment (b). The data showed in the graph is the mean ± SD of one experiment performed. Pit area measurements are also given as scatterplots for the number of pits scanned (c). Experiments were repeated three times with three different osteoclast preparations. (d) Immunoblotting analyses: equal amount of lysate proteins (15*μ*g) made from osteoclasts untreated or treated with peptides in the presence of TNF-*α* and bone particles for 12-14h was used for immunoblotting analyses with TRAP (top panel) and cathepsin K (Cath K; middle panel) antibodies. Immunoblotting with a GAPDH antibody was used as a loading control (bottom panel). Experiments were repeated twice.

*(i) Resorption Pit Formation Assay*. Resorption pits were significantly reduced in osteoclasts transduced with P1 peptide or added with NP1 (Figure 7(a)) which corresponds with the decrease in the number of NSZs and sealing rings (Figure 6). The pit areas were measured, and data were assembled from three slices per treatment. The data shown are the mean ± SD of one experiment performed (Figures 7(b) and 7(c)). The scattered plot in Figure 7(c) shows the area for each pit. A clear inhibition is apparent in osteoclasts treated with P1 or NP1.

*(ii) Immunoblotting with Antibodies to Cathepsin K and TRAP*. To further confirm whether the decrease in resorption is due to a decrease in the levels of specific resorption markers, we did immunoblotting analyses with antibodies against cathepsin K and TRAP (Figure 7(d)). The Western blot results demonstrated that all study group samples expressed a single band at ~37kDa for TRAP and ~24kDa for Cathepsin K. There are no significant differences in the expression levels of TRAP or cathepsin K between the study groups (Figure 7(d); lanes 1-5).

## 4. Discussion

The results obtained with NP1 demonstrate not only the uptake of the NPs by osteoclasts but also the delivery and biological effects of the peptide of interest. Results obtained with NP1 are consistent with the results of TAT-fused P1 peptide. Polymeric nanoparticles have been widely used for controlled delivery, biodegradability, biocompatibility, ease of surface modifications, and their ability to deliver a wide range of payload [29, 30, 45–50]. PLGA-NP as a peptide carrier was shown to induce a powerful response in vivo in mice or rat models [24, 49, 51, 52]. The results from these studies demonstrated either inhibition of osteoclastic differentiation or signaling pathway required for osteoclastic bone resorption [47, 48, 53]. Still, more investigations are needed to improve osteoporosis therapy as bone loss is seen in over 200 million people worldwide. It is highly essential to identify different targeting therapy and constant delivery of drug of interest to reduce either osteoclast differentiation or function. Here, we show that NP1 had no effect on adhesion of osteoclasts to dentine surface but inhibited the formation of sealing rings. Our results suggest the promising potential of PLGA-based nanocarriers as delivery systems for LPL peptide of interest to inhibit osteoclast function in vivo in mouse models. The sustained release of peptides for several days may possibly avoid injection of mice every day with peptides.

**Figure 8:**
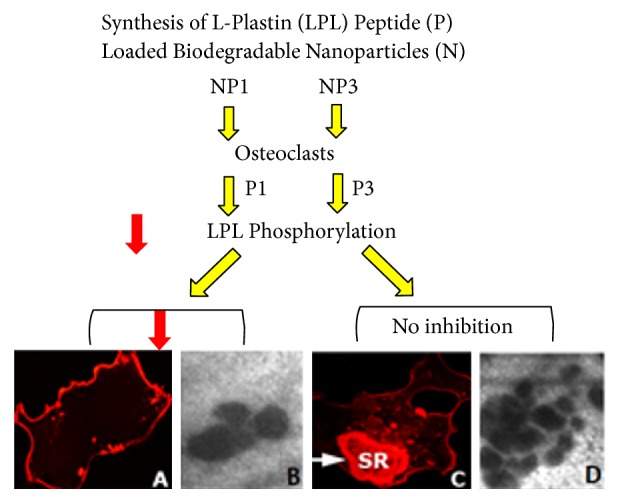
*Schematic representation of the effects of small MW L-plastin (LPL) peptides on osteoclast bone resorption*. Biodegradable and biocompatible PLGA-PEG nanocarriers were used to deliver and release the small molecular weight L-plastin peptides (NP1 or NP3) of interest in a controlled and sustained fashion. Osteoclasts were incubated with these nanoparticles of interest in the presence of TNF-*α*. The released peptide P1 attenuated TNF-*α* induced phosphorylation of cellular LPL. Therefore, the formation of NSZs and sealing rings as well as resorption activity are significantly reduced (red arrows) in osteoclasts in vitro. No inhibition was found with the control peptide (P3). Sealing ring (SR) is indicated with an arrow in C.

Although several targeted therapies are currently available to treat and/or prevent osteoporosis by blocking osteoclast activity, evidence shows that long-term treatments cause a reduction in osteoblast-mediated bone formation, resulting in atypical skeletal fractures [32, 54]. An ideal therapeutic scenario would be the one that impairs osteoclast function without interfering with osteoblast-driven bone formation. We have recently shown that TAT-fused LPL peptides did not inhibit the formation of bone by osteoblasts. Plastin 3 and not LPL (aka plastin 2) is expressed in osteoblasts. Mutations in plastin 3 resulted in osteoporosis in mice. This signifies the role of plastin 3 in osteoblasts and not osteoclasts [55]. Inhibition of resorption and not bone formation by P1 peptide highlighted the essentiality of LPL in osteoclast actin remodeling [9]. Here, we showed attenuation of formation of NSZs, sealing rings, and resorption pits in osteoclasts treated with NP1 and not NP3 validated the importance of LPL phosphorylation in the regulation of actin modulation involved in the formation of NSZs. These observations suggest that peptide-loaded PLGA NPs (NP1 and NP3) (a) have no toxicity on differentiated osteoclasts as NPs have no effect on viability or adhesion because osteoclasts displayed peripheral podosomal structures; (b) are bioactive. Bioactive nature of NP1 is apparent in the inhibition of phosphorylation of endogenous LPL in a competitive manner; hence the formation of NSZs, sealing rings, and resorption by osteoclasts are reduced. However, these changes are not observed with NP3 (Schematic Figure 8). We have identified a novel regulatory role for LPL in osteoclasts. It seems indispensable in the process of actin bundling processes involved in the formation of NSZs.

The treatment of bone loss is largely based on the use of agents that blocks bone resorption by osteoclasts (e.g., bisphosphonates, estrogens, SERMS, and calcitonin). Estrogen deficiency is associated with increased osteoclast activation, decreased osteoblast function, and increased inflammatory bone-resorbing cytokines such as interleukin-6 (IL-6) and TNF-*α*. Estrogen replacement therapy (ERT) in postmenopausal women is known to reduce bone loss; however, increased breast cancer risk along with other side effects still raises concerns following its use. Bone loss is also controlled with anti-TNF-*α* therapy. Unfortunately, only a limited number of patients are responsive to this treatment [56–59]. Recent studies indicated that systemic anti-TNF-*α* therapy prevents bone loss in mice through distinct mechanisms involving decreased bone resorption. However, several disadvantages have been reported including the cost of treatment, suppression of immune response, and the onset of neurologic symptoms [60]. We have used the traditional osteoclast culture model system to provide a good rationale for using PLGA-PEG-NP1 as an antiresorptive therapeutic alternate for osteoclast-mediated bone loss (Figure 8).

The important limitation in this paper is the dearth of* in vivo* studies in animal models. To further this goal, our future study will focus on the effects of sNT-LPL peptides on bone remodeling in aging and ovariectomized mice models which demonstrate bone loss. However, handling mice for the daily injection of peptides is challenging. Therefore, we recognized the importance of developing a sustained peptide release system. Controlled and sustained drug delivery has been recognized as one of the most advanced research areas, and several strategies have been used to accomplish such systems. Nanoparticles coated with PEG have been shown to reduce phagocytosis and complement activation, as well as prolonged circulation time in the blood [51, 61–63]. We believe that this peptidomimetic nanotherapeutic design will provide new avenues to target actin modulation involved in osteoclast bone resorption without affecting osteoclast survival and adhesion to bone surfaces* in vivo*. Since osteoblasts do not express LPL, sNT-LPL peptide seems novel peptidomimetic therapeutics for bone loss* in vivo*.

## 5. Conclusions

We evaluated the perspective of peptide-loaded PLGA nanoparticles as peptidomimetics in reducing osteoclast function systematically without affecting podosome assembly or adhesion. A decrease in the formation of NSZs and sealing rings could be connected well with a decrease in the resorption activity of osteoclasts transduced with P1 or treated with NP1. P1 or NP1 had no inhibitory effect on the expression of bone resorption markers. We believe nanoparticles loaded with LPL peptide might provide a promising basis for the development of drug delivery systems influencing osteoclast activity. Peptides released from the PLGA nanoparticles had the biological activity in decreasing osteoclast cytoskeletal reorganization and bone resorption via decreasing the phosphorylation of LPL. Targeting LPL is our ultimate interest because of its role in the early phase of sealing ring formation which is crucial for osteoclast bone resorption. These* in vitro* findings are central for the forthcoming in vivo studies with mice models (aging and ovariectomized) demonstrating bone loss.

## Figures and Tables

**Table 1:** Physicochemical characterization of nanoparticles.

Nanoparticles	Size (nm)^a^	PDI^b^	Zeta potential (mV)^c^	Peptide loading (%)
PLGA-PEG_P1	157 ± 4	0.058 ± 0.02	- 3.6 ± 0.8	5
PLGA-PEG_P3	136 ± 5	0.062 ± 0.03	- 3.1 ± 0.2	5

Physicochemical characterization data represents the average of 3 independent experiments ± SD.

^a^ Hydrodynamic diameter (number mean) measured by dynamic light scattering.

^b^ Polydispersity index (PDI) indicates the distribution of individual molecular masses in a batch of nanoparticles, measured by dynamic light scattering.

^c^ Surface charge measured at 25°C in 15x diluted PBS with ~9 mM NaCl, pH 7.4.

Table 1 layout is used from Wadajkar et al., 2017 (ref. [20]) (under the Creative Commons Attribution License/public domain).

## Data Availability

The data used to support the findings of this study are included within the article.

## Abbreviations

ABP: Actin-binding protein
RANKL: Receptor activator of NF-*κ*B ligand
M-CSF: Macrophage colony stimulating factor
LPL: L-Plastin
sNT-LPL: Small molecular weight amino terminal L-plastin peptide
NT-LPL: Amino-terminal L-plastin peptide
TNF-*α*: Tumor necrosis factor alpha
TAT: Transactivator peptide with transforming properties
PLA: Poly(l-lactide) acid
PLGA: Poly(l-lactide-*co*-glycolic) acid
PCL: Poly €-caprolactone
NP1: Nanoparticle loaded with P1 peptide
NP3: Nanoparticle loaded with P3 peptide
BMP-2: Bone morphogenic protein-2
PEG: Polyethylene glycol
NSZs: Nascent sealing zones.

## Additional Points

*Highlights*. (i) Biodegradable PLGA-PEG nanoparticles (N) loaded with small molecular weight peptidomimetics (inhibitory (P1) and control (P3)) of L-plastin demonstrated successful cellular uptake and release in osteoclasts in vitro. (ii) The inhibitory P1 peptide released from NP1 reduced significantly the formation of sealing rings and hence resorption of dentine matrix by osteoclasts in vitro. (iii) Results strongly support the possibility of using this nanoparticles strategy as therapeutics for bone loss.
